# Supplementary material for: Quantitative approaches to variant classification increase the yield and precision of genetic testing in Mendelian diseases: the case of hypertrophic cardiomyopathy
Source: Genome Med. 2019 Jan 29;11:5. doi: 10.1186/s13073-019-0616-z (PMC6350371; doi:10.1186/s13073-019-0616-z)
Supplement: Supplementary file 2 — Supplementary figures S1 and S2. (DOCX 742 kb) [file 13073_2019_616_MOESM2_ESM.docx]

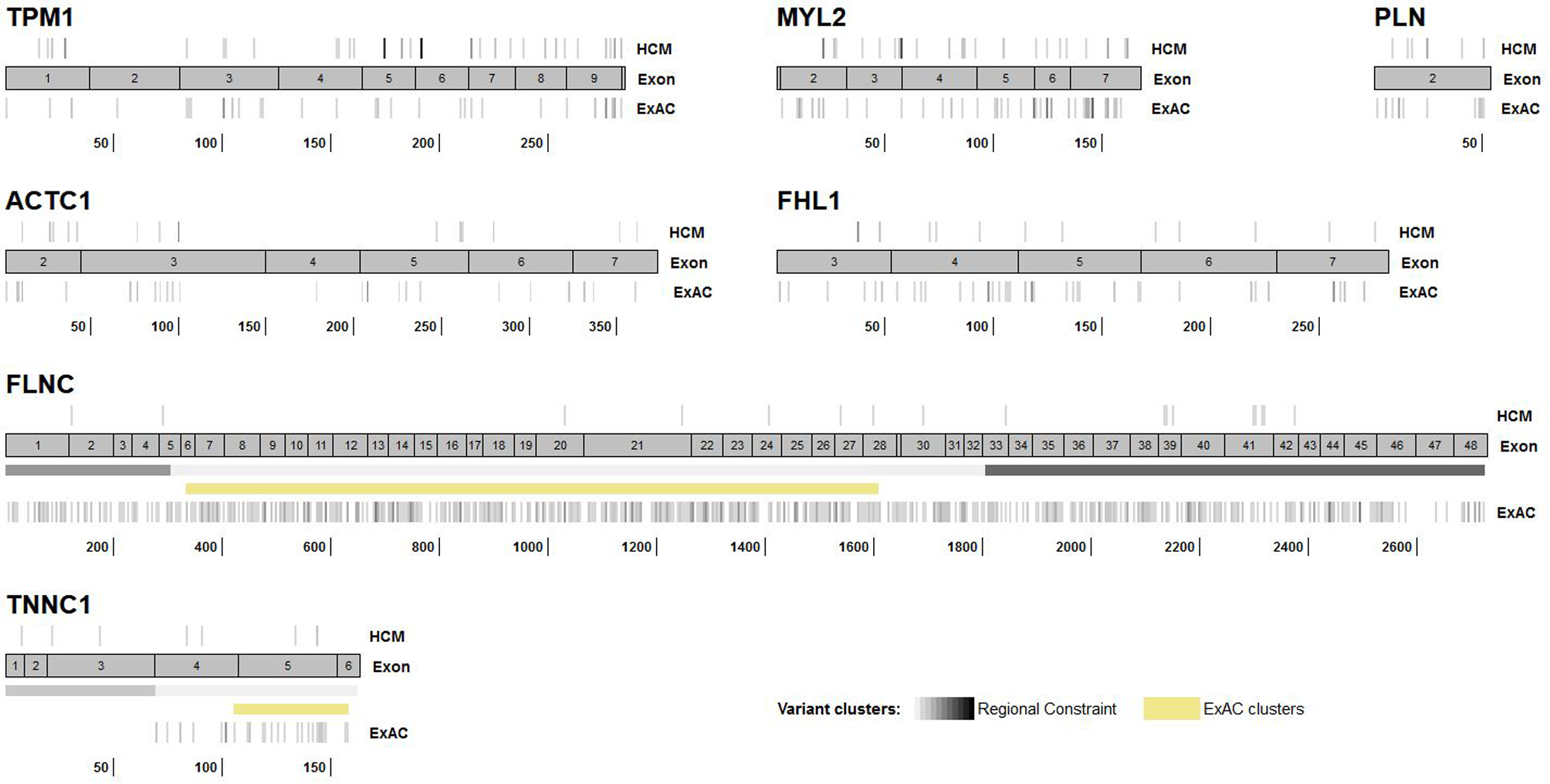


**Figure S1: Distribution of rare variants in HCM and ExAC cohorts in genes without variant clustering**

*Figure S1: Distribution of rare, non-truncating variants in HCM cohorts and ExAC for validated genes without observed clustering of variants in cases (variant density increases with darker shades of grey). Regional constraint boundaries described by Samocha* et al *are also highlighted.*


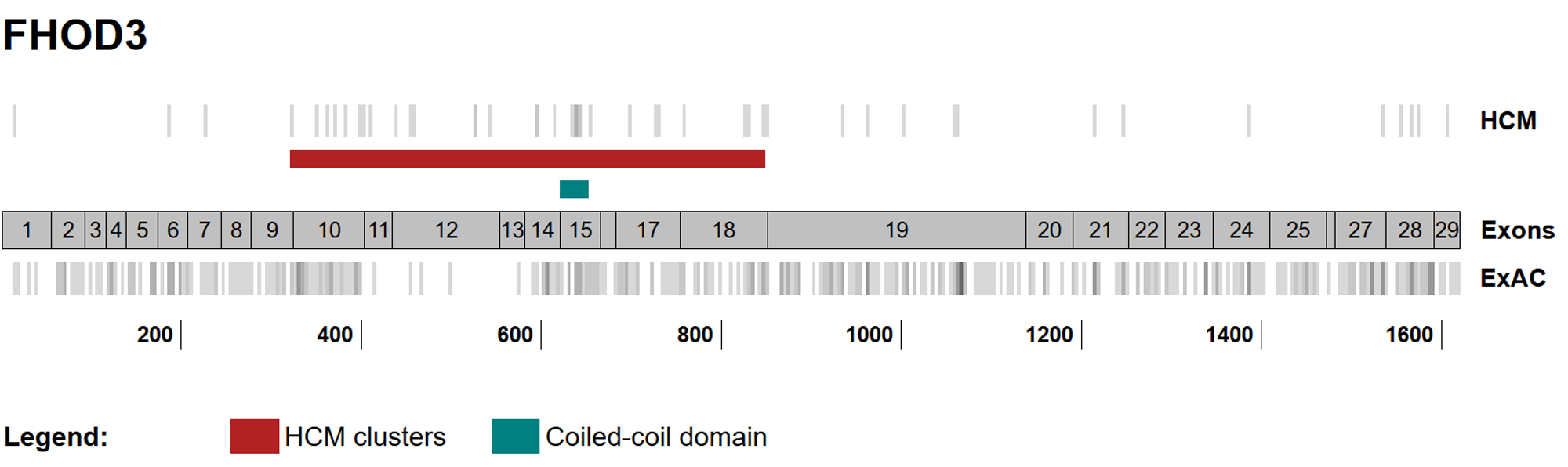


**Figure S2: Distribution of rare variants in HCM and ExAC cohorts in FHOD3**

*Figure S1: Distribution of rare, non-truncating variants in HCM cohorts and ExAC for the recently implicated HCM gene FHOD3. An enriched case cluster was detected between residues 321 and 849. The EF for rare, non-truncating variants within this cluster is 0.786 (0.726-0.847) compared to 0.475 (0.353-0.597) for the overall gene. The coiled-coil domain (residues 622-655) is also highlighted – clustering of cases variants in this domain has been observed, with a calculated EF of 0.823 (0.709-0.937).*
